# Computerized Childbirth Monitoring Tools for Health Care Providers Managing Labor: A Scoping Review

**DOI:** 10.2196/medinform.6959

**Published:** 2017-06-15

**Authors:** Michael S Balikuddembe, Nazarius M Tumwesigye, Peter K Wakholi, Thorkild Tylleskär

**Affiliations:** ^1^ Center for International Health University of Bergen Bergen Norway; ^2^ Department of Epidemiology and Biostatistics Makerere University Kampala Uganda; ^3^ Department of Obstetrics & Gynaecology Mulago National Referral and Teaching Hospital Kampala Uganda; ^4^ School of Public Health Department of Epidemiology & Biostatistics Makerere University Kampala Uganda; ^5^ School of Computing & Informatics Technology Makerere University Kampala Uganda

**Keywords:** childbirth, obstetric labor, fetal monitoring, medical informatics applications, systematic review

## Abstract

**Background:**

Proper monitoring of labor and childbirth prevents many pregnancy-related complications. However, monitoring is still poor in many places partly due to the usability concerns of support tools such as the partograph. In 2011, the World Health Organization (WHO) called for the development and evaluation of context-adaptable electronic health solutions to health challenges. Computerized tools have penetrated many areas of health care, but their influence in supporting health staff with childbirth seems limited.

**Objective:**

The objective of this scoping review was to determine the scope and trends of research on computerized labor monitoring tools that could be used by health care providers in childbirth management.

**Methods:**

We used key terms to search the Web for eligible peer-reviewed and gray literature. Eligibility criteria were a computerized labor monitoring tool for maternity service providers and dated 2006 to mid-2016. Retrieved papers were screened to eliminate ineligible papers, and consensus was reached on the papers included in the final analysis.

**Results:**

We started with about 380,000 papers, of which 14 papers qualified for the final analysis. Most tools were at the design and implementation stages of development. Three papers addressed post-implementation evaluations of two tools. No documentation on clinical outcome studies was retrieved. The parameters targeted with the tools varied, but they included fetal heart (10 of 11 tools), labor progress (8 of 11), and maternal status (7 of 11). Most tools were designed for use in personal computers in low-resource settings and could be customized for different user needs.

**Conclusions:**

Research on computerized labor monitoring tools is inadequate. Compared with other labor parameters, there was preponderance to fetal heart monitoring and hardly any summative evaluation of the available tools. More research, including clinical outcomes evaluation of computerized childbirth monitoring tools, is needed.

## Introduction

In 2015, an estimated 303,000 women died from pregnancy-related complications such as excessive bleeding, obstructed labor, and infections [1,2]. The obstructed labor complex directly contributes to 6-10% of the maternal deaths, in addition to contributing to other diseases for the mother and the baby [3,4].

Proper monitoring of the labor and delivery process with appropriate action based on findings is one of the keys to the prevention of pregnancy-related diseases and deaths [5,6]. Labor monitoring includes three main areas, namely fetal conditions, labor progress, and maternal conditions. The fetal parameters include fetal heart rate and amniotic fluid color, whereas labor progress is tracked through cervical dilation, uterine contractions, and fetal descent. The parturient’s condition is monitored by her blood pressure, temperature, urine, and mental state. The monitoring in many low-resource settings is hampered by the lack of user-friendly tools for labor management, limited access to evidence-based clinical guidelines for the providers of maternal health services, maternity provider factors, weak referral networks, and limited health financing [7].

Since 1994, the paper partograph has been promoted by the World Health Organization (WHO) as the standard labor monitoring tool [8], but to date its use is still poor in many low-resource settings due to many user and usability challenges [1,9-11]. To address these challenges, scientists in maternal health called for improvements of the partograph [1,12,13]. The WHO called for the development and evaluation of pragmatic electronic health (eHealth) solutions to health challenges [14].

Noteworthy, mobile health (mHealth) was embraced in many settings, especially chronic conditions with concomitant improvement in medical care [15,16]. It was hoped that next-generation system innovations could improve the quality of care during childbirth and reduce maternal deaths [17,18]. However, there seems to be a paucity of papers on computerized labor monitoring tools as is with mHealth in general.

We had a notion that the responses to various calls for better labor monitoring tools are still poor. Therefore, we set out to determine the volume and scope of research on computerized monitoring tools that can be used by health care providers in childbirth management.

## Methods

We undertook a scoping review, as defined elsewhere [19,20], to assess the reactions to the WHO call for labor monitoring tools (computerized or otherwise) with the potential of being more acceptable to the stakeholders in maternity services. Tricco et al (2016) reiterate the purpose of scoping reviews as, “…to present a broad overview of the evidence pertaining to a topic, irrespective of study quality, and are useful when examining areas that are emerging, to clarify key concepts and identify gaps” [19]. In June and July 2016, we searched PubMed and Google Scholar databases for peer-reviewed and gray literature. The search was supplemented by manual searches in Google search engine and ResearchGate online repositories for other papers meeting the selection criteria.

The inclusion criteria were a paper written in English language, addressing an aspect of a computerized or mobile labor monitoring tool, for use by maternity service providers, and written between January 1, 2006 to May 31, 2016. We excluded literature on tools for use primarily by expectant parents and the numerous apps for nonprofessional use such as contraction monitoring at home or fetal growth monitoring.

Our key terms in the search included “labor,” “monitoring,” “computer,” “mobile,” “tool,” “provider,” “delivery,” and “birth.” We combined them in various ways to get search strings. An example of such a string is “With all ‘labor monitoring’ + plus at least one of ‘computer$ tool$ mobile provider$ delivery birth - consumer’ (anywhere in article).” At the title review stage, we combined the term “labor” with one or more of the other terms to get relevant papers.

Data collection started with the individual researcher or research assistant identifying and screening papers for allocated years. We then entered the search terms and filtered the results according to the desired years of publication. We sorted the results in ascending years to ease tracking of the viewed Web pages. Each collector exported the identified papers into Mendeley-1.16.1 reference manager (by Elsevier) and used it to remove duplicates.

The data collector imported the Mendeley (by Elsevier) output into Google Scholar or PubMed and applied more specific filters to the titles. The filters helped eliminate the nonhuman birth-related and non-computer tools. The subsequent titles were saved to an online library for further screening. Abstracts to the saved titles were downloaded and qualitatively analyzed to determine the target users. Uncertainty about the eligibility of an article was consensually resolved based on the selection criteria.

**Figure 1:**
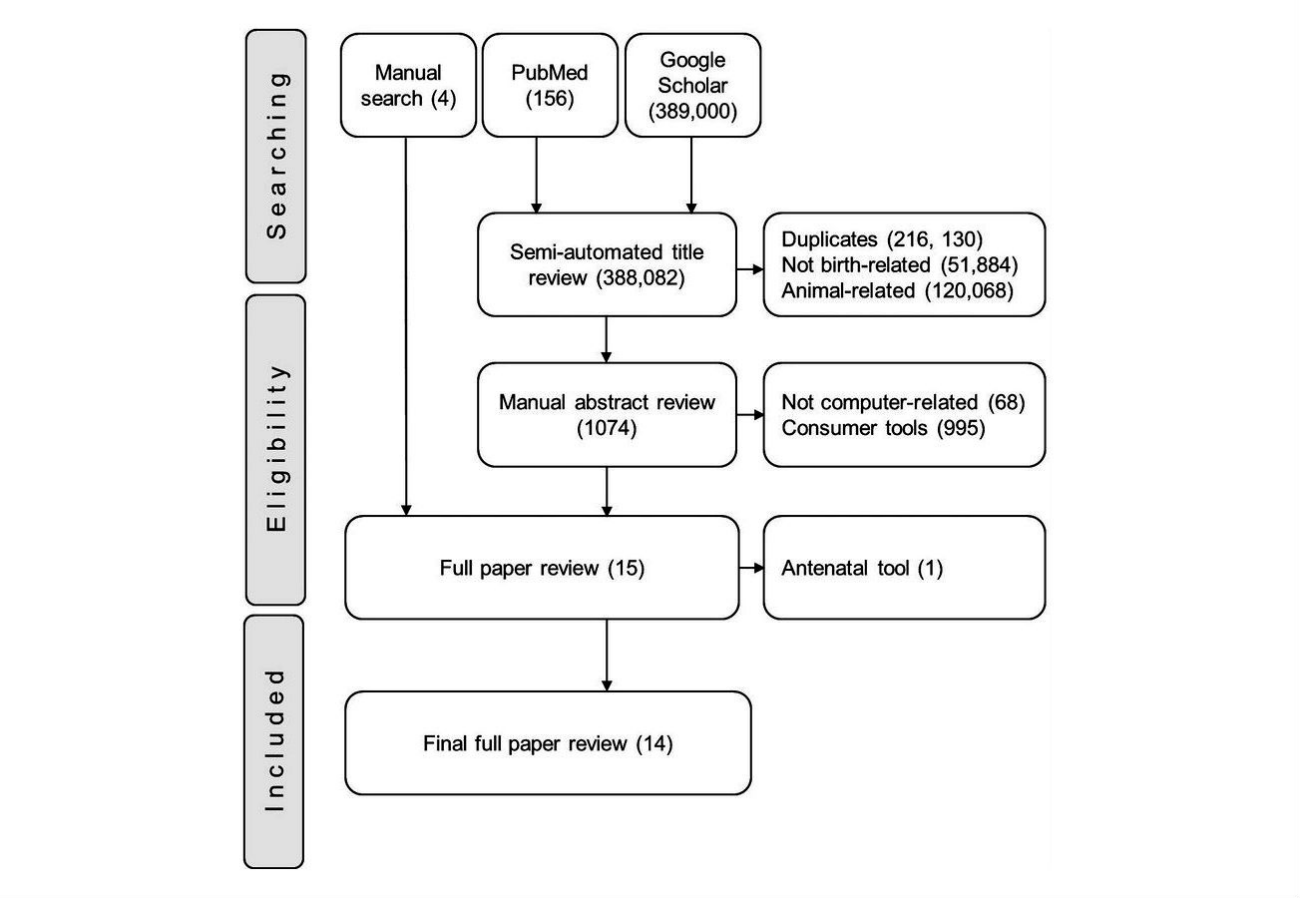
The steps of paper selection depicted in a PRISMA flow diagram. The number of articles included or excluded at each step is shown in brackets.

Full papers to abstracts with all eligibility criteria were retrieved. Manually identified abstracts or papers that were not part of the controlled search were added to the pool. All papers were scrutinized against all selection criteria. The remaining papers were included in the quantitative final analysis. These steps are summarized, as shown in Figure 1 *,* to mimic the preferred reporting items for systematic reviews and meta-analyses (PRISMA). Each paper was read to decipher the focus, developmental stage, and computing platform of the labor monitoring tool.

## Results

### Volume of Relevant Research

In the preliminary literature search, we retrieved 389,000 titles from Google Scholar and 156 from PubMed (Figure 1). These were imported into Mendeley 1.16.1 (by Elsevier) and duplicates removed. The titles were semiautomatically reviewed to remove papers about nonhuman or nonbirth related tools, which resulted in the exclusion of 388,082 entries. During the screening of abstracts for the remaining 1074 papers, we eliminated papers about tools that did not incorporate computers during labor monitoring use to leave 11 abstracts. Four abstracts identified in the manual literature search were added to the remaining 11 to get 15 abstracts used to identify papers for full review. At the full paper review stage, one paper (the Bacis program study by Horner in South Africa) was excluded because its subject tool was not designed for use during labor, which left 14 papers with all inclusion criteria for final analysis, as shown in Table 1.

### Stage of Tool Development

As shown in Table 2, 4 out of 14 analyzed papers addressed labor monitoring tools still at the planning phase. We analyzed 7 papers about tools that had reached the implementation stage. The authors of the remaining 2 papers addressed the cost and impact on clinic workflow evaluation of one tool, QUALMAT eCDSS [21].

Five tools that were in the clinical trial or field testing [22-25] phase were classified under the implementation stage of development. Three tools had undergone formative assessment in form of user or cost evaluations [23,26,27]. We retrieved literature on a tool that was designed and tested in 2007, but we did not get publications on its advancement. One tool had a summative evaluation of its cost and impact on clinic workflow [28]. We did not get any tool with definitive summative evaluation, that is, pragmatic clinical outcomes (morbidity or mortality) studies.

### Focus of Labor Monitoring Tool

The main parameters of focus in the analyzed papers were monitoring all labor parameters (7 papers) and fetal heart (3 papers). Four tools were designed to monitor one of three labor monitoring sections, whereas the rest could monitor multiple sections, as shown in Table 3. Authors addressing multiple labor monitoring sections were chiefly concerned with electronic versions of the partograph computing abilities. Therefore, their innovations potentially addressed the 15 parameters on a partograph. Those on fetal status focused on fetal heart monitoring with a cardiotocogram (CTG). The authors who addressed labor progress only specifically targeted cervical dilation and descent or station of a cephalic fetus.

**Table 1:** List of papers included in the final analysis and the name of study tools.

Paper (Reference number)		Year^a^	Name of tool
“Quality of prenatal and maternal care: Bridging the know-do gap” (QUALMAT study): an electronic clinical decision support system for rural sub-Saharan Africa [21]		2013	QUALMAT eCDSS^b^
The Moyo fetal heart rate monitor [22]		2014	Moyo monitor
mLabour: design and evaluation of a mobile partograph and labor ward management application [23]		2016	mLabour
Continuous monitoring of cervical dilatation and fetal head station during labor [24]		2007	Computerized labor-monitor
The design and implementation of the PartoPen maternal health monitoring system [25]		2013	PartoPen
Improving maternal labor monitoring in Kenya using digital pen technology: a user evaluation [26]		2012	PartoPen
Cost-effectiveness of a clinical decision support system in improving maternal health care in Ghana [27]		2015	QUALMAT eCDSS
Impact of an electronic clinical decision support system on workflow in antenatal care: the QUALMAT eCDSS in rural health care facilities in Ghana and Tanzania [28]		2015	QUALMAT eCDSS
A mobile multi-agent information system for ubiquitous fetal monitoring [29]		2014	Fetal IMAIS
A study of an intelligent system to support decision making in the management of labour using the cardiotocograph–the infant study protocol [30]		2016	INFANT
The development of a simplified, effective, labour monitoring-to-action (SELMA) tool for better outcomes in labour difficulty (BOLD): Study protocol [31]		2015	SELMA
Life curve mobile application: an easier alternative to paper partograph [32]		2015	Life curve
ePartogram: a mobile decision support tool to address labor complications [33]		2013	ePartogram
Another set of eyes: Remote fetal monitoring surveillance aids the busy labor and delivery unit [34]		2010	ANGEL shield

^a^Year of publication.

^b^eCDSS: electronic clinical decision support system.

**Table 2:** Stages of development for the computerized labor monitoring tools.

Tool	Stage of development^a^				
	Plan	Designing	Implementing	Formative evaluation	Summative evaluation
QUALMAT eCDSS^b^	y^c^	y	y	y	z
Moyo monitor	y	y	z^c^	z	x
mLabour	y	y	z	y	x
Computerized labor-monitor	y	y	z	x	x
PartoPen	y	y	y	y	x
Fetal IMAIS	y	x^c^	x	x	x
INFANT	y	x	x	x	x
SELMA	y	x	x	x	x
Life curve	y	x	x	x	x
ePartogram	y	y	z	x	x
ANGEL shield	y	y	y	z	x

^a^Based on the five stages of software development.

^b^eCDSS: electronic clinical decision support system.

^c^x: stage not reached, y: completed stage, z: stage incomplete.

**Table 3:** Parameters on modified WHO partograph noted in the capability of computerized tools.

Parameter on WHO partograph	Computerized tool to monitor parameter										
	21^a^	22^b^	23^c^	24^d^	25^e^	29^f^	30^g^	31^h^	32^i^	33^j^	34^k^
Fetal heart	y^l^	y	y		y	y	y	y	y	y	y
Cervix opening	y		y	y	y		y	y	y	y	
Descent of leading part	y		y	y	y		y	y	y	y	
Amniotic fluid	y		y		y		y	y	y	y	
Molding of head	y		y		y		y	y	y	y	
Uterine contractions	y		y	y	y		y	y	y	y	
Maternal pulse	y		y		y		y	y	y	y	
Maternal blood pressure	y		y		y		y	y	y	y	
Maternal temperature	y		y		y		y	y	y	y	
Urine protein	y		y		y		y	y	y	y	
Urine acetone	y		y		y		y	y	y	y	
Urine volume	y		y		y		y	y	y	y	
Time of membrane rupture	y		y		y		y	y	y	y	
Drug given	y		y		y		y	y	y	y	
Clinical diagnosis suggestion	y	y	y		y		y	y	y	y	

^a^QUALMAT eCDSS.

^b^Moyo monitor.

^c^mLabour.

^d^Computerized labor-monitor.

^e^PartoPen.

^f^Fetal IMAIS.

^g^INFANT.

^h^SELMA.

^i^Life curve.

^j^ePartogram.

^k^ANGEL shield.

^l^Parameter can be monitored with the tool.

**Table 4:** Computing platforms and adaptability for the labor monitoring tools.

Tools	Computing Platform				Adaptable
	Portability	Network environment	Operating systems	Software base	
QUALMAT	Laptop and desktop	Stand-alone	MS Windows	Java	Yes
Moyo monitor	Mobile	Stand-alone	Customized	Undisclosed	No
mLabour	Mobile	Client-server	Android	Application	Yes
Computerized labor-monitor	Mobile and desktop	Stand-alone	Nonspecific	Ultrasound waves	Unknown
PartoPen	Mobile	Stand-alone	Livescribe pen	LiveCode “Penlet”	No
Fetal IMAIS	Mobile & desktop	Client-server, wireless	MS Windows	Java	Yes
INFANT	Mobile and desktop	Offline	MS Windows	Undisclosed	Unknown
SELMA	Mobile and desktop	Offline	Not applicable	Undisclosed	Unknown
Life curve	Mobile	Client-server	Android	Application	Yes
ePartogram	Mobile	Client-server	Android	Undisclosed	Unknown
ANGEL shield	Desktop	Client-server	MS Windows	Undisclosed	Yes

All tools were intended for clinical diagnosis support based on algorithms. The least basic was the capability to take measurements or give alerts and reminders to maternity care providers [24,29,34]. However, 8 of 11 tools could also provide diagnosis and action suggestions to the user. The proposed SELMA tool could use machine learning models to predict diagnosis and outcomes for different contexts of use.

### Computing Platforms for the Labor Monitoring Tools

As shown in Table 4 *,* the tools were mostly usable on existing personal computer hardware, especially mobile gadgets such as phones, laptops, and desktop computers. Majority communicated through client-server networks, and 4 of 11 (36%) used stand-alone computers. Microsoft Windows was the most commonly used operating system in desktops, and Android was used in most mobile systems. One tool (PartoPen) uses custom-made software, whereas the application-based tools were developed in a Java environment. For half of the tools, the authors did not specify the software framework used in development. Of 11 tools, only 4 were reported as customizable to suit a user’s context of work.

The authors of the analyzed papers planned for adaptable tools. The intended context of tool use was stated as low-resource settings apart from the ANGEL shield. This computer program was made for and operated in a university hospital, but there were plans of rolling it out to nearby rural health centers. For 5 of 11 tools, it was reported that they could be customized to different contexts of use and even more functions added, where necessary.

## Discussion

### Principal Findings

In this review, out of over 380,000 papers, 14 qualified for the final analysis. They represented studies of 11 computerized tools capable of aiding health workers in maternity care. All labor parameters could be monitored by 7 of the 11 tools upon implementation. Only one tool had summative evaluations, which included cost and indicator studies. We did not find evidence of morbidity or mortality evaluation for any tool. Most tools used open source software that was also adaptable to common computers.

In this study, we chose to conduct a scoping review due to an ostensibly low volume of systematic evaluations and publications but with potentially more works on the subject. To this effect, many papers on pregnancy care applications were identified, although they were excluded from analysis for failure to meet other inclusion criteria. PubMed and Google Scholar formed the basis for the main search results due to their popularity among authors and a wide coverage of subjects. They were augmented with a manual search that indeed yielded more papers [22,23,32,33] included in the final analysis. The inclusion period of 2006 to 2016 was chosen to encompass the 5 years before and after the 2011 WHO call to evaluate all design and scale-up stages in eHealth [14], including e-labor monitoring tools.

Many papers were identified, which echo the findings of Kortteisto et al (2014), that is, mHealth is widespread [16]. However, similar to WHO and International Confederation of Midwives concerns in 2011 [13,14], only a handful addressed maternal labor and delivery monitoring. Moreover, as Hall et al found [17], the authors reported on tools that were in developmental stages, protocol preparation to field testing, without the definitive summative evaluation (pragmatic clinical outcomes studies) needed in health care research [35].

This state of affairs may be due to the human-intensive nature and high litigation potential of labor monitoring events that designing and testing a reliable computerized labor tool calls for more effort than is needed for an average medical condition. Another factor that could deter innovators is the difficulty in definitive summative evaluation of the tools in light of many confounders of labor outcomes. A similar situation in 2011 could have led the WHO to call for better research and evaluation of mHealth [14] such as labor monitoring tools. One should also be cognizant of the diverse labor monitoring contexts both within and without health facilities and communities, which were highlighted in the 2016 Lancet maternal health series [36,37]. However, the trend of research seems to be in a positive direction. From the works analyzed in this paper, the majority of the papers are authored after the WHO call to action, and all the documented evaluations were published after the call.

After the data collection for this review, another call to action on improving quality of maternity and newborn care was sounded through the Lancet maternal health series [37]. Furthermore, the potential of mHealth to help out is anticipated, with innovators urged not to be stifled by the fear of liability and litigation but to provide evidence-based tools for woman-centered care [18,38]. This series reinforced the findings of this review and stressed the need for further research for context-specific mobile childbirth monitoring tools.

Regarding the focus of the tools, it was obvious that the three main sections—fetal, labor progress, and maternal states—of labor monitoring received unequal attention. Monitoring the fetal condition, especially the fetal heart, was most researched, perhaps due to the discovery of cardiotocogram (CTG) that is efficacious in detecting fetal distress. CTG is sonoelectric and improving it through computerization was easier than inventing tools as was necessary for the labor progress. On the other hand, cervical dilation and maternal conditions are subjectively dependent on provider skills, and sociocultural or religious norms. Hence, the diversity of these conditions could hamper the design, testing, and development of widely acceptable tools to monitor labor progress and maternal conditions. With the recognition of the diversity of contexts of use but limited resources, it is prudent that generic tools are designed and developed or adapted for specific contexts [39].

The operating system platforms were generic in about half of the tools perhaps to cut development costs. This is also good for the diverse hardware that is increasingly portable. On the other hand, the programming software base was mostly undisclosed. This could be due to the early stages of development. Moreover, the authors were not yet committed to a specific program. However, the generic Java development environment was used in most specified cases. This would suggest that even the rest are more likely to use it in tool development.

### Limitations

The sources of data for this review were not exhaustive of all literature (eg, papers not written in English), and as such, we could have omitted some papers on the computerized labor monitoring tools. This is a drawback, but the main sources are wide enough for an adequate sample and similar reviewers [17,20,40] increasingly adopt this approach. We also started the search with broad terms and narrowed them down as we saved different papers for subsequent analyses.

### Conclusions

In conclusion, the scope and volume of research on computerized maternal labor monitoring tools is likely to be narrow and small. Fetal heart monitoring seems to dominate over other labor management parameters. Most tools are designed to use affordable computing platforms, but there is hardly any summative evaluation of the available tools. This may imply a slow response to the call for developing and evaluating computerized labor monitoring tools that could reduce labor-related disease. Further research, including clinical outcomes studies and publication of results, is needed on computerized tools for use in comprehensive childbirth monitoring.

## Abbreviations

CTG: cardiotocogram
eCDSS: electronic clinical decision support system
eHealth: electronic health
mHealth: mobile health
PRISMA: preferred reporting items for systematic reviews and meta-analyses
WHO: World Health Organization
